# MicroRNAs as Prognostic Biomarkers for Atrial Fibrillation Recurrence After Catheter Ablation: Current Evidence and Future Directions

**DOI:** 10.3390/biomedicines13010032

**Published:** 2024-12-26

**Authors:** Emmanouil P. Vardas, Evangelos Oikonomou, Panos E. Vardas, Dimitris Tousoulis

**Affiliations:** 1 1st Cardiology Department, General Hospital of Athens “Hippokration”, University of Athens Medical School, 11528 Athens, Greece; vardas.man@gmail.com; 2 Department of Cardiology, General Hospital of Athens “G. Gennimatas”, 11527 Athens, Greece; 3 3rd Cardiology Department, Sotiria Regional Hospital for Chest Diseases, University of Athens Medical School, 11527 Athens, Greece; 4 Biomedical Research Foundation Academy of Athens, Heart Sector, Hygeia Hospitals Group, Attica, 15123 Athens, Greece

**Keywords:** atrial fibrillation, fibrosis, inflammation, electrical remodeling, microRNA, recurrence

## Abstract

Atrial fibrillation (AF) is the most common cardiac arrhythmia encountered in clinical practice and is associated with significant morbidity and mortality. Even though catheter ablation has emerged as an available and effective treatment for AF, recurrence remains a significant challenge. This review presents the existing evidence on the prognostic role of microRNAs (miRNAs) in the prediction of AF recurrence after catheter ablation. We examined studies investigating the association between miRNA expression and post-ablation AF recurrence. Multiple miRNAs have been highlighted as potential biomarkers, which are involved in pathophysiological processes such as atrial remodeling, fibrosis, and inflammation. Despite some promising results, there has been significant heterogeneity across the studies. In this review, we demonstrate the potential miRNAs that can be routinely used as biomarkers of AF recurrence, and we identify areas that require further research to validate their clinical utility.

## 1. Introduction

Atrial fibrillation (AF) is the most common sustained cardiac arrhythmia that affects millions of people worldwide and is associated with an increased risk of stroke, heart failure, and mortality. According to the Global Burden of Disease 2019 study, the number of individuals living with AF worldwide rose from 33.5 million in 2010 to 59.7 million in 2019, representing a dramatic increase over just one decade. The prevalence of AF is projected to continue rising, with estimates suggesting 15.9 million cases in the United States by 2050 and 17.9 million cases in Europe by 2060 [1,2].

Catheter ablation, particularly pulmonary vein isolation (PVI), has emerged over the last few years as an effective treatment for AF, especially among patients who are refractory to antiarrhythmic drugs. However, AF recurrence after catheter ablation remains one of the most significant challenges, as recurrence rates range between 20% and 40% within the first year.

Predicting AF recurrence after catheter ablation could be crucial for patient selection and management in order to improve outcomes. Commonly assessed clinical factors such as age, left atrial size, and AF subtype have demonstrated a limited predictive value [3,4,5]. In recent years, there has been growing interest in the potential of microRNAs (miRNAs) to be used as biomarkers for AF recurrence. MicroRNAs are small, non-coding RNA molecules that play a crucial role in post-transcriptional regulation of gene expression. Multiple studies have demonstrated that miRNAs are implicated in various cardiovascular processes, including cardiac remodeling, fibrosis, inflammation, and arrhythmogenesis [6,7,8,9]. MiRNAs have been proven attractive candidates as biomarkers due to their stability in circulation and tissue-specific expression patterns [10,11,12]. This review aims to synthesize current evidence on the prognostic role of miRNAs in AF recurrence following catheter ablation. We will explore various miRNAs studied in this context, their potential mechanisms of action, and their predictive value for AF recurrence.

Inevitably, aiming to identify the prognostic significance of miRNA for AF recurrence post-ablation, we needed to broaden our study and the objectives of this review, exploring more broadly the role of miRNAs in the inflammatory and fibrotic processes that are frequently responsible for the development of arrhythmia or its relapses post-ablation [13].

From the outset, it is necessary to emphasize the complex nature of these pathophysiological pathways, which are most commonly characterized by the interplay of multiple actors.

## 2. Current Predictive Methods for AF Recurrence Post-Ablation

Several predictive models and scoring systems have been developed to assess the risk of AF recurrence following ablation procedures. (Table 1) Traditional models of risk estimation are based on the well-known conditions that lead to the development of atrial fibrillation, possibly with a different weighting of significance among the various proposed scores.

The HASBLP score, which incorporates AF history (H), age (A), snoring (S), body mass index (B), anteroposterior diameter of the left atrium (LA), and persistent AF (P), demonstrated satisfactory predictive performance, with an area under the curve (AUC) of 0.7668 (0.7298–0.8037), specificity of 73.47%, sensitivity of 67.73%, and accuracy of 71.69% [14].

This model has shown greater efficacy in predicting AF recurrence during a 2-year follow-up compared to the conventional risk stratification system CHADS2 (congestive heart failure, hypertension, age > 75, diabetes, and prior stroke/TIA) and CHA2DS2 scores (congestive heart failure, hypertension, age > 75, diabetes, prior stroke/TIA, vascular disease, age 65–74, and sex) scores [15,16].

Other predictive models such as DR-FLASH (diabetes mellitus, renal dysfunction, persistent form of atrial fibrillation, left atrial diameter, age, female sex, and hypertension), CAAP-AF (coronary artery disease, left atrial diameter, age, presence of persistent or long-standing atrial fibrillation, number of antiarrhythmic drugs failed, and female sex), ATLAS (age, type AF, LA volume, sex and smoking), APPLE (age, persistent AF, impaired eGFR, LA diameter, EF), and MB-LATER (male, bundle branch block, left atrial diameter, type of AF, and early recurrent AF) have also shown encouraging results in relation to their studies. It should be noted, however, that while on the univariate analysis, all scores were significantly associated with low voltage areas (LVA), and on the multivariate analysis, only APPLE (OR 1.789, *p* < 0.001) and DR-FLASH (OR 2.144, *p* < 0.0001) remained significant predictors [17,18,19,20].

Moreover, another predicting tool for AF recurrence is the HATCH score, which incorporates hypertension, age, stroke/transient ischemic attack history, chronic obstructive pulmonary disease, and heart failure [21,22].

The BASE-AF2 (body mass index, left atrial diameter, smoking, early AF recurrence, duration of AF >6 years, and non-paroxysmal AF type) score, although developed initially for cryoablation patients, showed superior predictive ability in patients with concurrent AF and pulmonary diseases compared to other scoring systems. The optimal point for predicting AF recurrence of the BASE–AF2 score in the ROC analysis was 1 point with a sensitivity of 69.03% and specificity of 60.21% [23,24,25]

This score incorporates several factors, such as previous heart failure, current smoking status, and early AF recurrence—all of which have been identified as significant predictors of late AF recurrence. More recent predictive modeling attempts include the Atrial Fibrillation Ablation Recurrence Score (AFA-Recur), a machine-learning-based probability score that has realized good predictive performance for 1-year risk of recurrent atrial arrhythmia post-AF ablation [26,27].

As research in this field continues to change, the integration of several predictive factors at the clinical, electrophysiological, and molecular marker levels will more than likely increase the accuracy of predicting AF recurrence [28].

Most of the currently presented predictive models are highly influenced by clinical factors, being unable to fully reflect complex pathophysiological processes involved in recurrence, such as atrial remodeling, fibrosis, and inflammation. Most of these models provide a static evaluation of recurrence risk, failing to take into consideration dynamic changes in either patient characteristics or the course of the disease over time. Moreover, predictive performance can be different for specific ablation techniques, further limiting generalizability across different clinical practices. Last but not least, most current methods do not give personalized enough risk estimates, which are important for tailoring post-ablation management to the individual patient. These limitations emphasize the need for more sensitive and dynamic biomarkers for the prediction of AF recurrence.

MicroRNAs—reflecting the different components of atrial remodeling, fibrosis, and inflammation—represent one of the most promising tools to reach improved risk stratification, allowing development of more individualized management strategies in patients with atrial fibrillation undergoing catheter ablation.

## 3. Methods

A comprehensive literature search was conducted over the following available databases: PubMed, Scopus, and Web of Science. The search terms used included various combinations of “microRNA”, “miRNA”, “atrial fibrillation”, “catheter ablation”, “recurrence”, and “prognosis”. We included original research articles that were published in English and investigated the association between miRNAs and AF recurrence after catheter ablation. Case studies and non-English publication were excluded from this review.

## 4. Results

### 4.1. MicroRNAs Involved in Atrial Remodeling and Fibrosis

Numerous studies, including in vitro experiments, animal models, and clinical research, have demonstrated that mRNA plays an essential role in atrial and channel remodeling and fibrosis—processes that are significant in AF pathogenesis and recurrence.

Selectively, certain significant research studies that examined the pathophysiological pathways and the role of specific miRNAs in the development of atrial fibrosis are presented as follows: Pradhan K. et al. in vitro studies investigated the role of miR-25 and particularly miR-21-5p in atrial remodeling by simulating AF conditions in cultured cardiomyocytes. It was demonstrated that miR-21-5p was released in vitro from cardiomyocytes under tachyarrhythmia conditions and stimulated fibroblasts in a paracrine manner to induce collagen production [29].

Furthermore, in animal models, van den Berg N.W.E. et al., in their systematic review analysis, highlighted the role of miR-21 in atrial fibrosis and miR-29’s association with structural remodeling [6].

Additionally, Zhou Q. et al. showed that plasma miR-21 levels are strongly correlated to left atrial low-voltage areas (LVAs), which are directly indicative of atrial fibrosis. Patients who exhibited lower miR-21 levels presented better outcomes after ablation, suggesting its potential to be used as a predictor and marker of ongoing atrial remodeling. Focusing on the molecular level, miR-21 promotes fibroblast survival and growth factor secretion by targeting Sprouty homolog 1 (SPRY1) and Phosphatase and tensin homolog (PTEN), which leads to enhanced fibrosis [30].

Along with the aforementioned, the findings of the study by Zhan J. et al. should also be taken into account, which demonstrated that the miR-29 family, and particularly miR-29b-3p, was also associated with atrial fibrosis and AF recurrence of the miR-29 family and particularly miR-29b-3p, has also been associated with atrial fibrosis and AF recurrence. These miRNAs regulate genes that are involved in extracellular matrix remodeling. Lower levels have been associated with increased left atrial diameter and higher AF recurrence rates. miR-29 directly influences the composition of the extracellular matrix by targeting multiple collagen isoforms, fibrillin, and elastin [31].

In addition to the aforementioned, it is important to consider that miR-30 and miR-133, which have also been implicated in the regulation of fibrosis, are downregulated in AF patients. miR-30 has been shown to target connective tissue growth factor (CTGF), while miR-133 regulates collagen 1A1 (COL1A1) and connective tissue growth factor (CTGF), both of which are key players in the development of fibrosis [32,33].

Using more simplistic wording, the previous findings show that in patients with AF, the factors miR-30 and miR-133, being downregulated, fail to downregulate CTGF and control further structural changes in the extracellular matrix of the myocardium in patients with atrial fibrillation.

**Figure 1:**
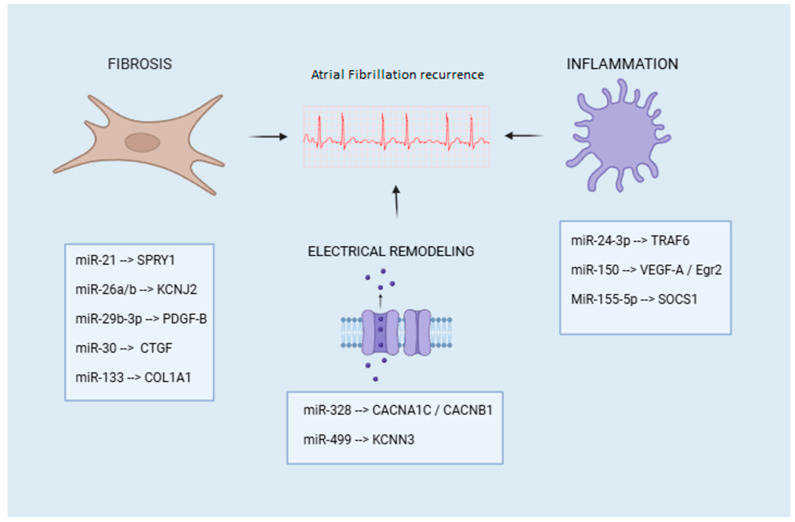
The figure illustrates how different miRNAs, along with their specific molecular targets, influence the recurrence of AF after catheter ablation via distinct pathophysiological mechanisms represented by fibrosis, inflammation, and electrical remodeling. SPRY1: Sprouty Homolog 1; KCNJ2: Potassium Inwardly Rectifying Channel, Subfamily J, Member 2; PDGF-B: Platelet-Derived Growth Factor Subunit B; CTGF: Connective Tissue Growth Factor; COL1A1: Collagen Type I Alpha 1 Chain; CACNA1C: Calcium Voltage-Gated Channel Subunit Alpha1 C; CACNB1: Calcium Voltage-Gated Channel Auxiliary Subunit Beta 1; KCNN3: Potassium, Calcium-Activated Channel Subfamily N Member 3; TRAF6: TNF Receptor Associated Factor 6; VEGF-A: Vascular Endothelial Growth Factor A; Egr2: Early Growth Response 2; SOCS1: Suppressor Of Cytokine Signaling 1.

In addition to the aforementioned, we would like to add to this synopsis that miR-26 molecules, such as miR-26a and miR-26b, have also shown interesting dynamics in relation to AF and ablation treatment. Dai Μ. et al. study demonstrated that the plasma levels of miR-26a and miR-26b were decreased in AF patients compared with the control group. Interestingly, the expression of miR-26a/b increased after the radiofrequency ablation (RFA) treatment, while it decreased the expression of P-selectin (SELP), a protein that has been studied as a mediator in inflammation and thrombosis. The aforementioned results show that RFA treatment may additionally affect AF by upregulating miR-26a/b, which in turn suppresses SELP expression. Taking a deeper look at the molecular level, miR-26 has been shown to target *KCNJ2*, which encodes the Kir2.1 subunit of the inward rectifier potassium current (IK1), influencing in that way the electrical properties of the cardiomyocytes [34] (Figure 1).

### 4.2. MicroRNAs Involved in Inflammation and Oxidative Stress

MiRNAs with a direct effect on inflammation processes have also demonstrated prognostic value in AF recurrence. In studies performed by McManus D. et al. and Vaze A. et al., plasma miR-150 showed lower levels in AF patients, which later increased after successful catheter ablation. Additionally, it was observed that a lower level of the aforementioned miRNA before catheter ablation was associated with an increased risk of AF recurrence during a one-year follow-up. miR-150 modulates angiogenesis and inflammatory responses by targeting vascular endothelial growth factor A (VEGF-A) and early growth response protein 2 (Egr 2), molecules directly related to the regulation of inflammation [35,36].

In view of the above, several therapeutic approaches could be adopted for the therapeutic modulation of miR-150 levels. One strategy that could be adopted involves miRNA mimics synthetic RNA molecules that are designed to mimic endogenous miR-150. Such mimics could be administered into cardiac tissue as a way to supplement the reduced miR-150 levels seen in patients with AF.

While these approaches are of theoretical interest, much remains to be learned about the complex role of miR-150 in AF pathophysiology and about how to safely and effectively deliver such potential therapies.

Furthermore, miR-155-5p and miR-24-3p, which have been associated with the regulation of endothelial nitric oxide synthase (eNOS) and nitric oxide (NO) signaling, were downregulated after the ablation procedure, suggesting that they could possibly influence the inflammatory processes that take place in the arrhythmogenesis of AF. In the study conducted by Wang M. et al., it was highlighted that these miRNAs might potentially be important in the modulation of the inflammatory component of AF pathogenesis. Specifically, it is hypothesized that miR-155 targets the suppressor of cytokine signaling 1 (SOCS1), which subsequently influences the Janus kinase/signal transducer and activator of transcription (JAK-STAT) pathway and inflammatory responses [37,38] (Figure 1).

### 4.3. MicroRNAs Related to Electrical Remodeling

Another critical aspect of AF pathophysiology is the electrical remodeling of the atrium, and several miRNAs have been linked to this process. Starting with experimental research studies, Jia X. et al. found that miRNA-1 accelerates right atrial tachypacing-induced atrial effective refractory period shortening by targeting potassium channels, specifically through the regulation of *KCNE1* and *KCNB2* gene expression [39].

Furthermore, other studies have demonstrated that miR-32 plays a role in calcium homeostasis and ion channel function. Its upregulation has been associated with increased AF susceptibility and a higher risk of post-ablation recurrence miR-328, which upregulation has been associated with AF susceptibility and post-ablation recurrence risk. miR-328 acts by directly targeting *Calcium Voltage-Gated Channel Subunit Alpha1 C (CACNA1C)* and *Calcium Voltage-Gated Channel Auxiliary Subunit Beta 1 (CACNB1)* genes, which encode the L-type Ca2+ channel’s α1c and β1 subunits, respectively, directly affecting the calcium handling in cardiomyocytes [40,41].

Another miRNA implicated in the electrical remodeling of the atrium, which could be potentially linked to AF recurrence, is miR-328, which contributes to the adverse atrial electrical remodeling in AF by targeting L-type Ca (2+) channel genes.

Moreover, the miR-499 is involved in the regulation of the small-conductance *calcium-activated Channel Subfamily N Member 3 (KCNN3)* gene, which encodes the SK3 channel, an important regulator of atrial repolarization. In the study of Chen X. et al., a work that aimed to identify miRNAs associated with chronic AF by bioinformatics and experimental validation, it was found that the expression level of miR-1 was increased, and miR-499 was decreased in the ventricular pacing group, correlated with left atrial fibrosis and apoptosis in AF [42] (Figure 1).

### 4.4. Novel MicroRNAs Associated with AF Recurrence After Catheter Ablation

Several miRNAs have been identified by recent studies to be potentially associated with AF recurrence after catheter ablation (Table 2).

In a study performed by Lage R. et al., miR-451 was correlated with early AF recurrence post-ablation. miR-451a expression was severely downregulated in the left atrium of patients with AF recurrence when compared with patients without relapse. Additionally, patients with higher pre-ablation plasma levels of miR-451a were associated with an increased risk of AF recurrence within 6 months from the procedure. The same study also confirms that an increased scar percentage in the left atrium is positively correlated with AF recurrence [43].

Furthermore, Harada M. et al. have shown that plasma miR-20b-5p and miR-330-3p could be used as cardiac-specific biomarkers of the progression of atrial remodeling as well as prognostic markers of AF recurrence after catheter ablation. The study demonstrated that the expression of these miRNAs was negatively correlated with the size of the left atrium, while also they were downregulated in patients who presented new episodes of AF compared to healthy subjects during a 1 year follow up [44].

Zhou Q. et al. attempted to correlate the levels of specific miRNAs as well as some electroanatomic elements with AF recurrence post-ablation. Specifically, the study showed that miR-21 was strongly correlated with low-voltage areas (LVAs) while also being associated with the outcome of the procedure. Lower serum miR-21 levels were associated with better outcomes and limitation of new AF episodes, while higher levels were associated with AF relapse [30].

The key finding of a study conducted by Liu et al. showed that miR-409-3p and miR-432 levels were significantly lower in AF patients when compared with the healthy control group. Interestingly, the levels of these miRNAs normalized after catheter ablation reaching similar levels to the control group. Additionally, researchers speculate that the downregulation of miR-409-3p in AF patients affects the *β-fibrinogen* gene mRNA levels, leading to increased fibrinogen levels, which could exacerbate the risk of thrombosis [45].

Similarly, in the studies conducted by Vaze A. et al., a group of miRNAs was identified as involved in the atrial remodeling in patients with AF. Specifically, circulating microRNAs, 106b, 26a-5p, 484, 20a-5p, were associated with atrial fibrillation [35,36].

The same group of authors, a couple of years before the aforementioned publication, had presented the results of the miRhythm study, where it was shown that plasma levels of miRNAs 125a-5p and 10b in patients with paroxysmal AF were 3-fold lower after ablation compared with pre-ablation (*p* < 0.01). Pre-ablation plasma expression of miRNAs 125a and 10b, as well as miRNAs 60, 30a-3p, and 199b, were higher among patients with an AF recurrence compared with those without recurrence after ablation (*p* < 0.05), even after adjustment for clinical risk factors.

On top of the aforementioned, Shen X.B. et al. investigated the potential association of the miR-125a rs12976445 polymorphism and post-ablation AF recurrence. The results suggest that lower levels of miR-125a promote AF relapse after catheter ablation while also the downregulation of this miR might be attributed to the presence of the rs12976445 polymorphism. Patients with the GG genotype had increased miR-125a levels and longer AF recurrence-free survival compared with those with GC/CC genotypes, suggesting that genetic variations in miRNA genes can influence recurrence risk. Shen et al. also suggest that miR-125a could play a crucial role in AF recurrence by targeting IL-6R, a pro-inflammatory factor that participates in the phosphorylation of the transcription factors STAT1 and STAT3, further enhancing the inflammatory processes [46].

Finally, Dai Μ. et al. highlighted the role of miR-26a and miR-26b in AF. These miRNAs were found to be downregulated in AF patients while also they showed a negative correlation with SELP expression, a cellular adhesion factor that is mainly expressed in platelets and endothelial cells that has a decisive role in the mediation of leukocytes and subsequently in the promotion of inflammation. Patients exhibited increased miR-26a/b levels and decreased SELP expression after RFA, suggesting a potential mechanism for the therapeutic effect of ablation [34].

Concluding the chapter on miRNA involvement in AF development and AF recurrences post-ablation, it is important to emphasize that recent research findings support the involvement of certain miRNAs in the processes of arrhythmia development. Indeed, several miRNAs have shown a consistent association with the pathophysiology of atrial fibrillation, which is confirmed by multiple studies. However, for some others, the findings are conflicting and often contradictory.

## 5. Clinical Implications

Progressively, and despite the conflicting questions raised by many studies, the proarrhythmic role of specific miRNAs has been documented, contributing to increased atrial myocardial fibrosis—a predominantly proarrhythmic condition—while also playing a role in the malfunctioning of Na and Ca ion channels. Speculating based on the above, we argue that the use of microRNAs as prognostic biomarkers before or after atrial fibrillation procedures could lead to favorable outcomes in managing AF.

In the pre-ablation phase of a patient with atrial fibrillation, it is possible that miRNA phenotyping could—at least in the future—determine specific ablative interventions that would enhance the outcome. Unfortunately, similar studies do not exist for the post-ablation period. Although still speculative, a number of actions, such as the following, could influence therapeutic practice by personalizing treatment for each individual patient.

Firstly, these biomarkers can enable better risk stratification whereby clinicians will identify those patients at high risk of recurrence of AF with higher precision. With an enhanced ability to predict, the treatment could become more personalized, which in turn might improve the patient outcome and overall resource utilization. For example, those patients who are classified as high-risk according to their miRNA profile may benefit from more aggressive post-ablation monitoring or adjuvant interventions to prevent recurrence. The miRNA biomarkers may support the selection of patients most likely to benefit from catheter ablation. By finding those patients with a low likelihood of recurrence, clinicians could more confidently recommend ablation procedures, thus giving them a higher success rate and satisfaction for the patients. On the other hand, in cases where the miRNA profiles are less favorable, indicating high recurrence rates, alternative modes of treatment might be contemplated for these patients, thus saving them from undergoing unnecessary procedures.

Moreover, miRNA biomarkers would contribute to the elaboration of new therapeutic approaches. As knowledge regarding the role of individual miRNAs in AF pathophysiology continues to expand, targeted therapies aimed at modulating these miRNAs could be developed. New adjunctive treatments to prevent post-ablation recurrence of AF may result or even new standalone therapies for AF.

The possibility of measuring miRNA biomarkers by simple blood tests is a very important practical advantage. This non-invasive approach could facilitate easier and more frequent monitoring of patients, thus enabling timely detection of impending recurrence and early intervention. Moreover, the ability to track changes in miRNA levels over time may provide valuable insights into the progression of atrial remodeling and the long-term effectiveness of ablation procedures.

## 6. Discussion

The primary purpose of this scoping review is to present the potential role of a number of miRNAs as prognostic biomarkers for AF recurrence after catheter ablation.

Certainly, in the presentation of various studies, our work broadens, also showcasing results and findings related to the wide range of the proarrhythmic actions of these small, non-coding RNA molecules capable of regulating the expression of target genes.

It is true that, despite the advancements in the identification of a number of miRNAs and the progress made in our understanding of their role in the development of atrial fibrillation or in the occurrence of AF recurrences in the post-ablation period, it seems that the unknowns related to the above are still more than the knowns.

Primarily, the causal relationship between miRNA levels and AF recurrence needs to be established, as well as the reproducibility of the findings. To satisfy these two prerequisites, further well-organized and properly standardized studies are required.

Focusing on reproducibility, which remains a significant concern regarding the reliability of various findings, some miRNAs demonstrate a reproducible association with AF recurrence, while others have produced conflicting results.

At this point, we could choose two illustrative examples of conflicting evidence, which inevitably undermine, to some degree, the prognostic value of the overall narrative. The research group of Zhou Q. et al. [30] reported five years ago that low serum miR-21 levels were associated with better outcomes in the post-AF ablation period. Two years later, Sieweke J.-T. et al. [47] found, conversely, that low miR-21 levels were associated with a higher presence of atrial fibrillation.

Similarly, lower pre-ablation levels of miRNA-150 were associated with an increased AF recurrence risk, according to the studies of McManus D.D. et al. [35] and Vaze A. et al. [36], whereas the research group of Liau N.P.D et al. [38], on the other hand, found decreased expression of miRNA-150 in AF patients compared with controls, without establishing a clear link to recurrence.

It is clear, as mentioned earlier, that further research work is required, primarily based on well-selected, homogeneous, large-scale population samples and standardized methodologies for miRNA measurements in agreement with cut-off values that can be applied in a clinical context. Additionally, because most published studies rely on circulating miRNA, the tissue-specific expression of miRNA and the relationship between the latter and circulating miRNA levels need to be studied.

In conclusion, focusing on prediction models for AF recurrence in the early or late post-ablation period, it is evident that studies and substantial research work are needed to explore the possibility of creating newer models that would combine the classic—well-known—prognostic markers with a series of miRNAs.

## 7. Limitations and Future Perspectives

Based on the findings of this review, it is clear that the world of miRNAs, which has recently started to be uncovered, requires significant further work in order to clarify many unclear areas and for these non-coding RNA molecules to gain clinical significance. The limitations that have been identified so far, as well as the prerequisites for more reliable results, could be summarized as follows:

- Large-scale, multicentric studies have to be conducted to confirm the prognostic value of many promising miRNAs and to determine the most powerful biomarkers in various populations. Most of the studies reviewed in the current analysis were performed with relatively small sample sizes, thus limiting their generalizability. In most instances, the choice of these sample sizes was based on the particular study contexts, available resources, and preliminary data that may be inadequate to provide the power needed to detect smaller but clinically significant effects. This limitation underlines the need for larger, more definitive studies to confirm these initial findings.
- Standardization of miRNA measurement methods is necessary for the comparability of studies. This includes the harmonization of sample collection, processing, and analytical methodologies.
- The different statistical tests applied in most of the studies were selected according to the distribution of data and the analytical objectives, which may affect the reproducibility and comparability of the results. It is thus crucial that future studies ensure that a clear rationale is fully elaborated for the statistical methods chosen. The type of statistical test used by each study should be theoretically justified in light of the data nature, tested hypotheses, and study design. This would increase methodological transparency, with statistical approaches being more appropriate and reproducible. However, different statistical methods can introduce variability in sensitivity and data handling, which are significant limitations that should be acknowledged. The current variability in statistical methods across studies complicates the reproducibility of findings and makes it difficult to draw definitive conclusions across studies. The lack of such harmonization makes the comparison of the results from different studies and the identification of clinically relevant cut-off values difficult to achieve.
- Longitudinal studies that entail multiple time points are necessary for describing how miRNA levels vary over time and how these changes are related to long-term risk in AF recurrence. Most have focused on either preablation miRNA levels or short-term post-ablation changes, whereas long-term dynamics of miRNA expression in the context of AF recurrence remain poorly understood. Future studies should investigate comprehensive risk-prediction models combining miRNA levels with traditional clinical risk factors. This could potentially lead to a further improvement of AF recurrence prediction accuracy and help guide personalized treatment strategies for patients undergoing catheter ablation. While many studies have demonstrated links between miRNAs and AF recurrence, mechanisms are usually not well understood. Further mechanistic in vitro and in vivo studies are warranted to clearly explain how these miRNAs affect the pathophysiology of atrial fibrillation. This could include studies on target genes of these miRNAs and their roles in atrial remodeling and arrhythmogenesis. The potential of miRNAs as therapeutic targets for preventing AF recurrence should be explored. This could include studies on miRNA mimics or inhibitors in animal models of atrial fibrillation. If successful, such approaches could lead to new therapies for preventing AF recurrence after catheter ablation. Finally, most studies have focused on known miRNAs. Thus, few unbiased approaches, like next-generation sequencing, may identify new miRNAs related to AF recurrence and lead to the discovery of new biomarkers and therapeutic targets. In conclusion, while miRNAs show much promise as prognostic biomarkers for atrial fibrillation recurrence post-catheter ablation, considerable work is yet to be performed before translation into clinical practice.

Summarizing the above, it is understood that while miRNAs show much promise as prognostic biomarkers for atrial fibrillation recurrence post-catheter ablation, considerable work has yet to be performed before translation into clinical practice. Future studies that address some of the challenges and opportunities from this review may lead to improved risk stratification and personalized treatment strategies for patients with atrial fibrillation.

## 8. Conclusions

This scoping review illustrates the potential of miRNAs as predictive biomarkers of AF recurrence following catheter ablation. Various miRNAs involved in vital processes, including atrial remodeling, fibrosis, and inflammation, have been identified as predictors of AF ablation outcomes. Despite major challenges related to reproducibility and clinical translation, the combination of miRNA biomarkers with other clinical parameters may enhance risk stratification and personalized treatment strategies for patients with AF undergoing catheter ablation. As our knowledge about the role of miRNAs in the pathophysiology of AF grows, these small RNA molecules might have an increasing impact on the management of this common and complex arrhythmia.

## Figures and Tables

**Table 1:** This table lists various scoring models used to predict atrial fibrillation (AF) recurrence following catheter ablation. Each score includes a detailed list of components tailored to assess specific aspects of AF recurrence risk.

Score Name	Components
APPLE	Age, persistent AF, impaired eGFR, LA diameter ≥ 43 mm, EF < 50%
CAAP-AF	Coronary artery disease, LA diameter, age, persistent/long-standing AF, number of antiarrhythmic drugs failed, female sex
BASE-AF2	BMI > 28 kg/m^2^, atrial dilatation > 40 mm, current smoking, early AF recurrence, AF duration > 6 years, non-paroxysmal AF
MB-LATER	Male sex, bundle branch block, left atrial diameter ≥ 47 mm, type of AF (persistent), early recurrence
HATCH	Hypertension, age > 75 years, stroke/TIA, chronic obstructive pulmonary disease, heart failure
C2HEST	CAD/COPD (1 point each), hypertension, elderly (age ≥ 75), systolic heart failure, thyroid disease
CHADS2	Congestive heart failure, hypertension, age ≥ 75, diabetes, prior stroke/TIA
CHA2DS2-VASc	Congestive heart failure, hypertension, age ≥ 75, diabetes, prior stroke/TIA, vascular disease, age 65–74, sex category (female)
DR-FLASH	Diabetes mellitus, renal dysfunction, persistent AF type, LA diameter > 45 mm, age > 65 years, female sex, hypertension
ATLAS	Age, type of AF (paroxysmal, persistent, long-standing persistent), left atrial diameter, antiarrhythmic drugs failed, female sex
HASBLP	AF history, age, snoring, BMI, LA diameter, persistent AF
AFA-Recur	Machine-learning-based model using 19 pre-procedural clinical variables

**Table 2:** This table presents an overview of various microRNAs (miRNAs) and their expression patterns in atrial fibrillation (AF), changes post-ablation therapy, and their association with recurrence rates.

MicroRNA	Expression in AF	Change After Ablation	Association with Recurrence
miR-21	Upregulated	Decreased in responders	Higher levels associated with recurrence
miR-150	Downregulated	Increased in responders	Lower pre-ablation levels associated with recurrence
miR-26a/b	Downregulated	Increased	Lower levels associated with recurrence
miR-328	Upregulated	Decreased	Higher levels associated with recurrence
miR-409-3p	Downregulated	Increased in responders	Lower levels associated with recurrence
miR-432	Downregulated	Increased in responders	Lower levels associated with recurrence
miR-483	Unclear	Unclear	Potentially associated with recurrence (needs validation)
miR-125a	Upregulated	Decreased	Higher levels associated with recurrence
miR-206	Upregulated	Unclear	Higher levels associated with early recurrence
miR-10b	Upregulated	Unclear	Higher levels associated with recurrence
miR-601	Upregulated	Unclear	Higher levels associated with recurrence
miR-30a-3p	Upregulated	Unclear	Higher levels associated with recurrence
miR-199b	Upregulated	Unclear	Higher levels associated with recurrence
miR-29b-3p	Unclear	Unclear	Correlated with left atrial diameter and recurrence
miR-155-5p	Upregulated	Decreased	Higher levels associated with recurrence
miR-24-3p	Upregulated	Decreased	Higher levels associated with recurrence
miR-451a	Upregulated	Unclear	Higher levels associated with early recurrence

## Data Availability

Data is contained within the article.
